# Using quantitative immunohistochemistry in patients at high risk for hepatocellular cancer

**DOI:** 10.18632/genesandcancer.220

**Published:** 2022-06-06

**Authors:** Sobia Zaidi, Richard Amdur, Xiyan Xiang, Herbert Yu, Linda L. Wong, Shuyun Rao, Aiwu R. He, Karan Amin, Daewa Zaheer, Raj K. Narayan, Sanjaya K. Satapathy, Patricia S. Latham, Kirti Shetty, Chandan Guha, Nancy R. Gough, Lopa Mishra

**Affiliations:** ^1^The Institute for Bioelectronic Medicine, The Feinstein Institutes for Medical Research and Cold Spring Harbor Laboratory, Department of Medicine, Division of Gastroenterology and Hepatology, Northwell Health, NY 11030, USA; ^2^Department of Surgery, The George Washington University, Washington, DC 20037, USA; ^3^Department of Epidemiology, University of Hawaii Cancer Center, Honolulu, HI 96813, USA; ^4^Department of Surgery, University of Hawaii, Honolulu, HI 96813, USA; ^5^Lombardi Comprehensive Cancer Center, Georgetown University Medical Center, Washington, DC 20007, USA; ^6^Department of Neurosurgery, Zucker School of Medicine at Hofstra/Northwell, Hempstead, NY 11030, USA; ^7^Sandra Atlas Bass Center for Liver Diseases and Transplantation, Department of Medicine, North Shore University Hospital/Northwell Health, NY 11030, USA; ^8^Department of Pathology, The George Washington University, Washington, DC 20037, USA; ^9^Division of Gastroenterology and Hepatology, University of Maryland, Baltimore, MD 21201, USA; ^10^Department of Radiation Oncology, Albert Einstein College of Medicine, Bronx, NY 10461, USA

**Keywords:** liver cancer, immunohistochemistry, diagnostic model, cirrhosis, transforming growth factor beta

## Abstract

Hepatocellular carcinoma (HCC) is the primary form of liver cancer and a major cause of cancer death worldwide. Early detection is key to effective treatment. Yet, early diagnosis is challenging, especially in patients with cirrhosis, who are at high risk of developing HCC. Dysfunction or loss of function of the transforming growth factor β (TGF-β) pathway is associated with HCC. Here, using quantitative immunohistochemistry analysis of samples from a multi-institutional repository, we evaluated if differences in TGF-β receptor abundance were present in tissue from patients with only cirrhosis compared with those with HCC in the context of cirrhosis. We determined that TGFBR2, not TGFBR1, was significantly reduced in HCC tissue compared with cirrhotic tissue. We developed an artificial intelligence (AI)-based process that correctly identified cirrhotic and HCC tissue and confirmed the significant reduction in TGFBR2 in HCC tissue compared with cirrhotic tissue. Thus, we propose that a reduction in TGFBR2 abundance represents a useful biomarker for detecting HCC in the context of cirrhosis and that incorporating this biomarker into an AI-based automated imaging pipeline could reduce variability in diagnosing HCC from biopsy tissue.

## INTRODUCTION

A third of the global cancer deaths are attributed to primary liver cancer [1]. Hepatocellular carcinoma (HCC) is the most common primary liver cancer. HCC due to viral hepatitis has been declining; however, the incidence of HCC has been increasing due to the global increase in metabolic risk factors, such as diabetes, obesity, and hyperlipidemia [1–3]. HCC is rarely detected in early treatable stages. Most HCC occurs against a background of cirrhosis [3, 4]. Biomarkers that predict HCC development in patients with cirrhosis are critical, not only for effective diagnosis but also for prognosis and understanding the molecular basis of disease for rational therapeutic intervention [5].

Currently, recommended surveillance methods for HCC in patients with cirrhosis is biannual abdominal ultrasound screening with or without serum α-fetoprotein (AFP) testing [2, 3, 6]. However, ultrasound has limited sensitivity (62% overall and 77% in non-obese individuals) for early detection of HCC [7, 8], and the sensitivity drops to ~21% in obese patients [9]. High circulating AFP concentrations correlate with tumor burden, metastases, overall survival, and post-transplant recurrence [10–16]. However, close to 40% of patients lack circulating AFP, limiting the utility of this protein as an effective biomarker of HCC [17].

In cases where non-invasive imaging results are ambiguous, HCC is diagnosed by histologic features of biopsy tissue. Accuracy of diagnosis is improved by immunohistochemical (IHC) analysis for established biomarkers, such as glypican 3 (GPC3), heat shock protein 70 (HSP70), and glutamine synthetase (GS) [18–20]. However, the molecular complexity of HCC presents challenges finding reliable biomarkers for all patients [21]. Furthermore, the diagnosis is based on the pathologist’s interpretation, which can vary depending on the location of the sample of tissue analyzed, sample processing, and pathologist. Especially in clinical studies with images from multiple institutions, an automated pipeline for image processing and analysis could reduce variability in diagnosing HCC from biopsy tissue.

Changes in the abundance of transforming growth factor β (TGF-β) pathway components are associated with liver disease, including chronic liver disease, viral hepatitis, fibrosis, and HCC [22–26]. Qualitative studies indicate that TGF-β increases in chronic liver disease [23], whereas variable differences in the receptor subunits TGFBR1 and TGFBR2 have been reported [24–26]. RNA analysis of tissue from patients with chronic hepatitis C, compared with tissue from patients with cholelithiasis, revealed an increase in *TGFB1* transcripts and a decrease in *TGFBR2* transcripts [25]. High-intensity staining for TGF-β1, TGFBR2, and SMAD1, SMAD2, or SMAD3 correlated with fibrotic disease severity and inflammation [26]. These differences may relate to the etiology of the liver disease or whether the patients were developing HCC in a background of cirrhosis. Thus, we proposed that changes in TGF-β receptors could be biomarkers of HCC and that quantification of the changes in TGFBR1 and TGFBR2 could be used to predict the presence of HCC in cirrhotic tissue.

In this study, we used immunohistochemical analysis to explore the diagnostic potential of TGFBR1 and TGFBR2 as biomarkers to differentiate HCC from cirrhosis. To overcome variability in patient populations and sample preparation, we used tissue samples from biorepositories at 3 institutions: George Washington University (GW), the University of Maryland (UMD), and the University of Hawaii (UH). Sensitivity analysis for each set of samples was performed to eliminate potential sub-group effects. Tissue samples were obtained from biorepositories of pathological specimens from patients with HCC or patients with cirrhosis without HCC. Pathological specimens in the biorepositories were collected from patients undergoing liver transplantation (LT), liver resection, or liver biopsy. We validated the findings using automated image analysis based on a model created with deep-learning algorithms and artificial intelligence (AI). Finally, we tested the diagnostic potential of TGFBR1 and TGFBR2 staining intensity individually and together by logistic regression modeling to determine sensitivity, specificity, and accuracy of the model at multiple thresholds.

## RESULTS

### TGFBR1 and TGFBR2 abundance in tissue slides from cirrhosis-only patients or patient with HCC

**Figure 1:**
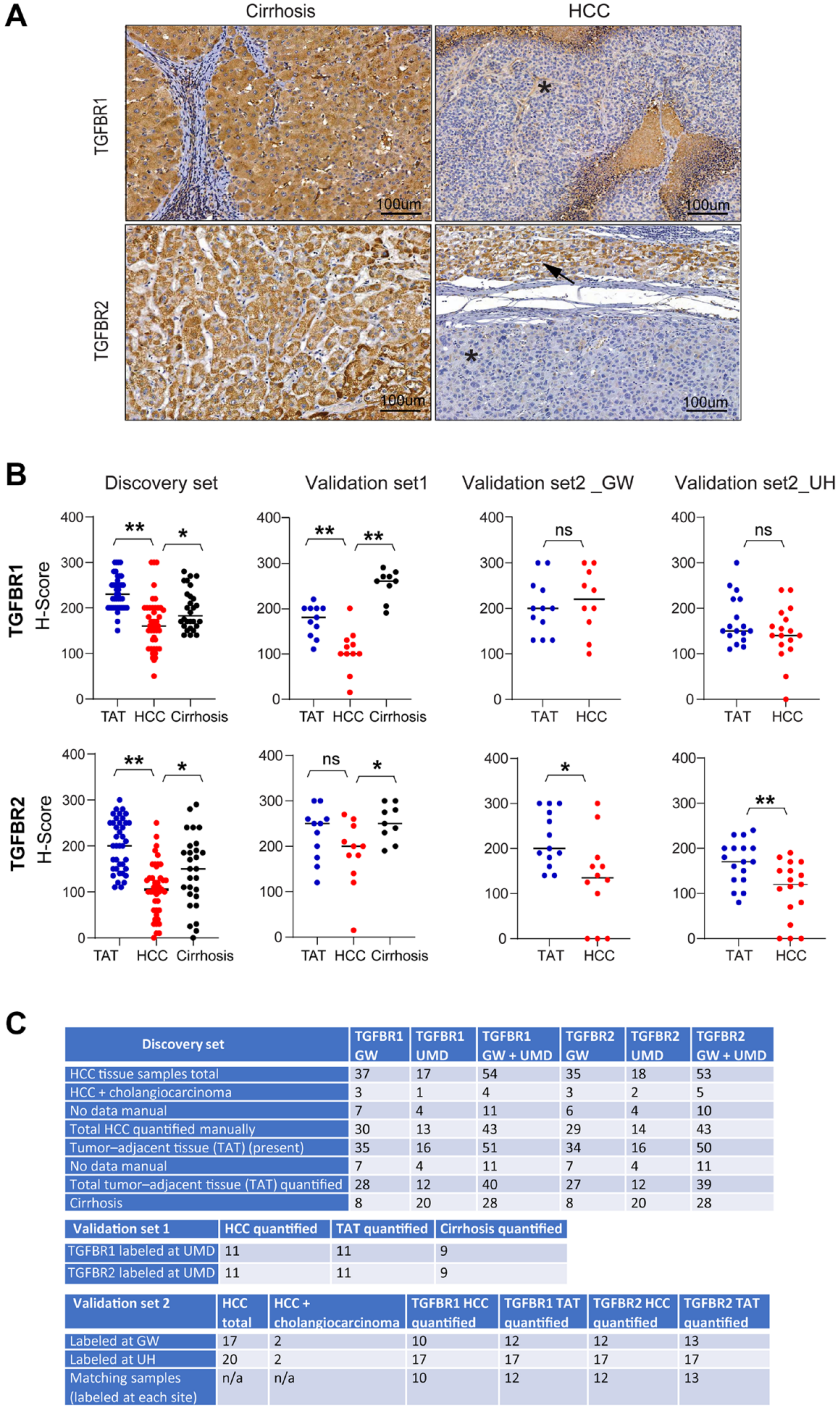
IHC analysis of TGFBR1 and TGFBR2 in liver tissue from cirrhotic patients with or without HCC. (**A**) Representative images of TGFBR1 and TGFBR2 labeling in patient tissue from GW. Asterisk marks tumor; arrow marks tumor-adjacent tissue (TAT). (**B**) H-score plots of TGFBR1 and TGFBR2 labeling intensity for the discovery set samples, validation set 1 samples, and validation set 2 samples. Samples in validation set 1 were from patients at UMD and were stained at UMD and evaluated at GW. Samples in validation set 2 were from patients at UH and were stained either at UH or GW and then were evaluated at GW. (**C**) Details of the samples in the discovery set, validation set 1, and validation set 2. Statistical significance between tumor-adjacent tissue and HCC and among tumor-adjacent tissue, cirrhosis and HCC were determined with two-tailed *t*-tests or one-way ANOVA (^*^p < 0.05; ^**^p < 0.005).

IHC staining intensities for TGFBR1 and TGFBR2 were lower in HCC tissue than in tumor-adjacent tissue (TAT) or in tissue from cirrhosis-only patients (Figure 1A). We manually quantified the intensity of staining for each protein by assigning H-scores to liver tissue samples collected from TAT, HCC, and cirrhosis in the discovery set from GW and UMD (Figure 1B and 1C). The mean and standard deviation (SD) of the H-score for TGFBR1 in HCC tissue (*n* = 43) were 165.0 ± 56.6, in TAT (*n* = 41) were 232.9 ± 36.6, and in cirrhosis-only tissue (*n* = 28) were 196.1 ± 43.8. For TGFBR2, the mean and SD were 105.8 ± 56.9 for HCC tissue (*n* = 43), 202.3 ± 55.5 for TAT (*n* = 40), and 145.2 ± 77.5 for cirrhosis-only tissue (*n* = 28). HCC tissue had a significantly reduced TGFBR1 H-score compared to TAT (*p* = 9.6 × 10^−9^) or cirrhosis-only tissue (*p* = 0.013). HCC tissue also had a significantly reduced TGFBR2 H-score compared to TAT (*p* = 3.6 ×10^−11^) or cirrhosis-only tissue (*p* = 0.028).

To validate these findings, we evaluated a separate set of samples from patients at UMD and that were labelled at UMD, validation set 1 (Figure 1B, 1C). The mean and SD for TGFBR1 H-scores were 106.4 ± 45.1 for HCC tissue (*n* = 11), 173.6 ± 33.4 for TAT (*n* = 11), and 252.8 ± 31.7 for cirrhosis-only tissue (*n* = 9). The reduced staining of TGFBR1 in HCC tissue was significant compared with that in TAT (*p* = 0.0013) or compared with that in cirrhosis-only tissue (*p* = 2.4 × 10^−7^). The mean and SD of the TGFBR2 H-scores were 183.6 ± 69.2 for HCC tissue (*n* = 11), 227.3 ± 55.6 for TAT (*n* = 11), and 251.7 ± 38.2 for cirrhosis-only tissue (*n* = 9). The difference of TGFBR2 staining intensity between HCC tissue and TAT was not significant (*p* = 0.14) but the difference between HCC tissue and cirrhosis-only tissue was significant (*p* = 0.018).

We evaluated a third set of samples, validation set 2, which were from patients at UH that were labeled at GW or UH (Figure 1B). The patients from UH all had HCC (Figure 1C). Therefore, only analysis of TAT and HCC tissue was possible. The mean and SD of the TGFBR1 H-scores were 216 ± 67.3 for HCC tissue (*n* = 10) and 202.5 ± 57.8 for TAT (*n* = 12) in the samples labelled at GW. For the samples labeled at UH, TGFBR1 H-scores were 143.5 ± 58.7 for HCC tissue (*n* = 17) and 172.9 ± 52.5 for TAT (*n* = 17). The differences in H- scores between HCC tissue and TAT for samples labelled at either location were not significant (*p* = 0.64 for those labeled at GW and *p* = 0.15 for samples labelled at UH). For TGFBR2, the mean and SD of the H-scores were 130.4 ± 93.3 for HCC tissue (*n* = 12) and 220.8 ± 58.6 for TAT (*n* = 13) for samples labelled at GW and 106.2 ± 63.3 for HCC tissue (*n* = 17) and 168.8 ± 46.9 for TAT (*n* = 17) for samples labelled at UH. TGFBR2 staining was significantly less in HCC tissue than in TAT labelled at either location (*p* = 0.013 for samples labelled at GW and *p* = 0.0034 for samples labelled at UH). Additionally, compared with the samples labelled at UH, the samples labelled at GW had higher mean H-scores for both TGFBR1 and TGFBR2 in both HCC tissue and TAT.

### Validation of TGFBR2 as significantly reduced in HCC by AI-based image analysis

**Figure 2:**
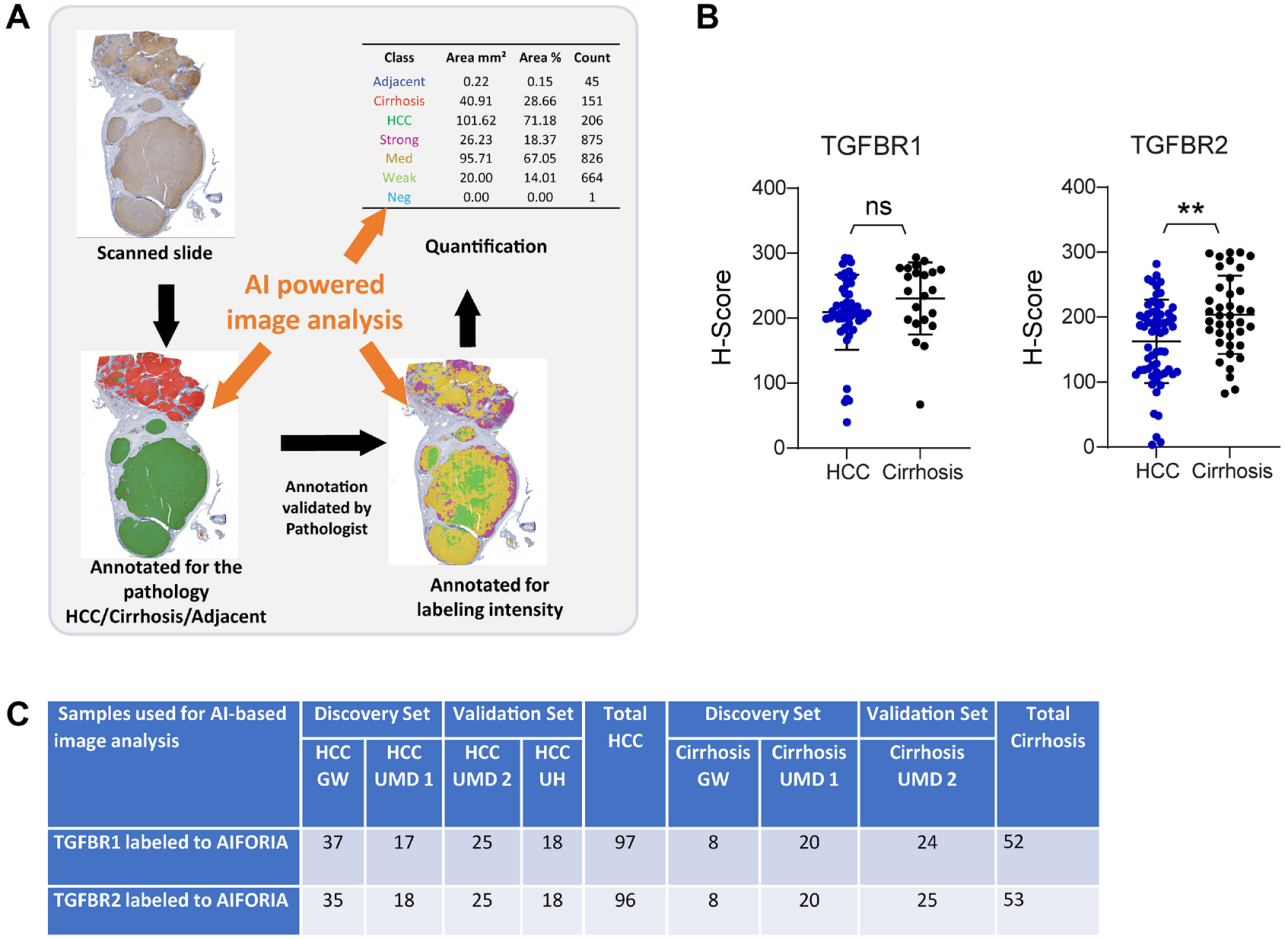
AI-based analysis of TGFBR1 and TGFBR2 staining intensity in HCC and cirrhotic tissue. (**A**) Overview of the workflow. (**B**) H-scores for TGFBR2 (HCC, *n* = 62; Cirrhosis, *n* = 39) and TGFBR1 (HCC, *n* = 50; Cirrhosis, *n* = 22) obtained by AI-based analysis. (**C**) Details of the samples provided for the AI-based analysis. Statistical significance was determined by two-tailed *t*-test (^**^p < 0.005).

As an independent validation approach, we used AI-based image analysis to automatically assign a H-score for TGFBR1 or TGFBR2 (Figure 2A–2C). After training the TGFBR1 and TGFBR2 algorithms on selected samples lacking large regions of necrotic tissue, H-scores were assigned using the algorithm on the remaining samples. A pathologist assessed the accuracy of each algorithm by comparing the AI-assigned H-score with a manually assigned score. This assessment indicated a higher error rate in the AI-based assignment of TGFBR1 values (3.08%) compared with TGFBR2 values (1.28%).

The mean and SD of the AI-assigned H-scores for TGFBR2 were 162.68 ± 63.66 in HCC tissue (*n* = 62) and 203.44 ± 59.33 in cirrhosis-only tissue (*n* = 39) (Figure 2B). This reduction of TGFBR2 intensity in HCC tissue was significant (*p* = 0.0017). In contrast, the mean and SD of the AI-assigned H-scores for TGFBR1 were 209.22 ± 57.14 in HCC tissue (*n* = 50) and 230.28 ± 54.2 in cirrhosis-only tissue (*n* = 22), and this was not a significant difference (*p* = 0.15) (Figure 2B).

### Evaluation of TGFBR2 staining intensity in patient-matched HCC tissue and tumor-adjacent tissue

We also compared the intensity of TGFBR1 and TGFBR2 staining in patient-matched samples of HCC and TAT (Supplementary Figures 1–3). Not all samples had scores for the receptors in both HCC and TAT (Table 1). Out of 69 samples with TGFBR1 staining in both sites, 74% had a lower H-score in HCC than in TAT. Out of 67 samples with TGFBR2 staining in both sites, 84% (56 out of 67) had a lower H-score in the HCC part of the sample. Using a higher threshold for the difference between the sites, we determined the paired samples that had a H-score for the TAT that was 10% higher than that in the HCC tissue. Using these more stringent criteria, 65% of the samples had higher TGFBR1 staining in the TAT and 78% had higher TGFBR2 staining. Of those samples that had a lower H-score in the HCC tissue (51 for TGFBR1 and 56 for TGFBR2), TGFBR1 staining in the TAT exceeded that in the HCC tissue by more than 10% in 45 (88%) of matched samples and TGFBR2 staining met this threshold in 52 (96%) of the matched samples.

**Figure 3:**
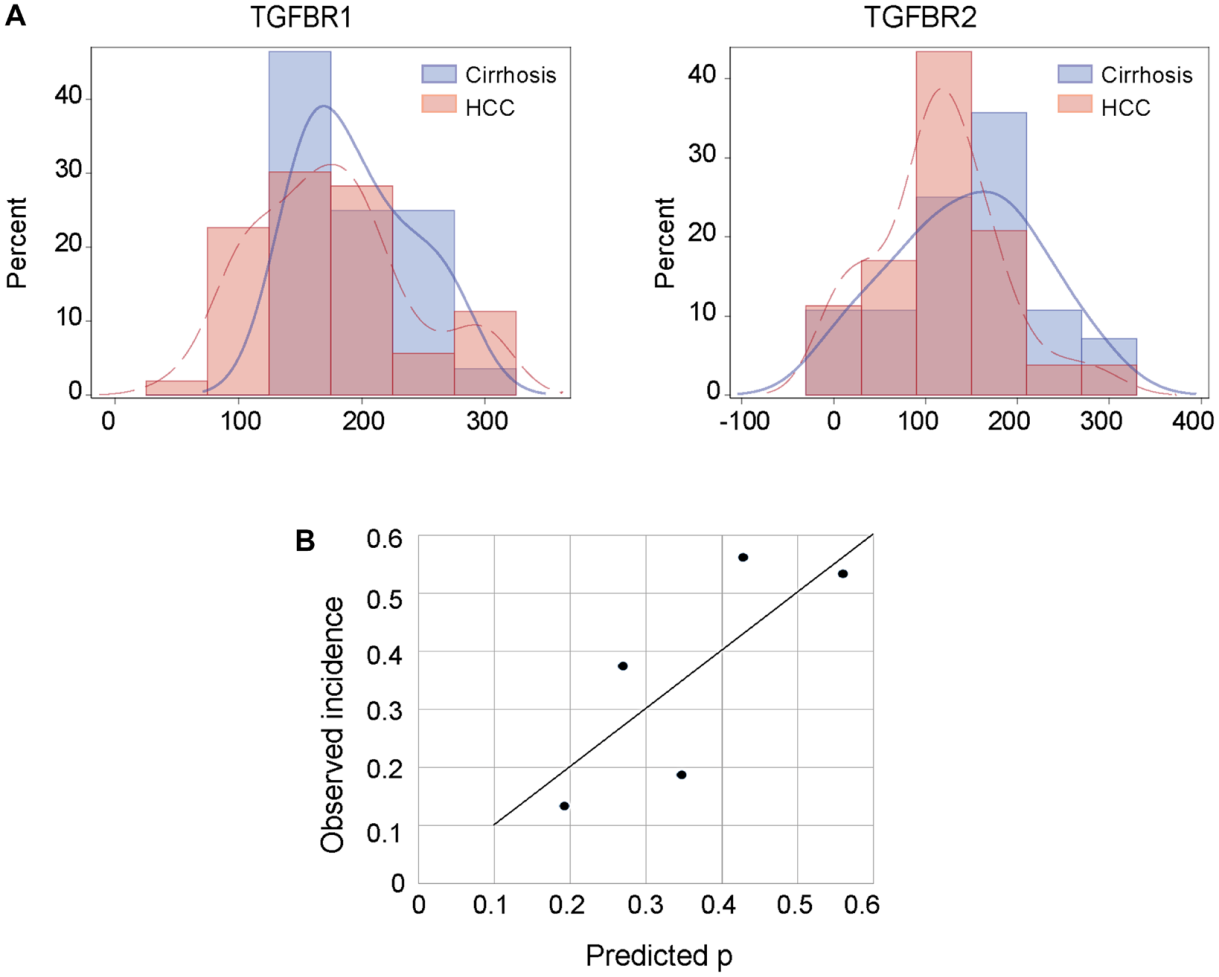
Development of diagnostic models for TGFBR1 and TGFBR2 labeling intensity in tissue sections. (**A**) Frequency histograms for H-scores of TGFBR1 and TGFBR2 stratified by diagnosis. Cirrhosis, blue; HCC, red. (**B**) Calibration of a logistic model including both TGFBR1 and TGFBR2 for predicting HCC versus cirrhosis.

**Table 1:** Analysis of TGFBR1 and TGFBR2 staining intensity in patient-matched HCC and tumor-adjacent tissue (TAT)

Samples	TGFBR1	TGFBR2
Total	85	83
Samples with both HCC and TAT H–scores	69	67
Samples with HCC < TAT	51	56
Samples with TAT 10% > HCC	45	52
Percentage of HCC < TAT out of samples with H-scores in both HCC and TAT tissue	74% of 69	84% of 67
Percentage of samples with TAT 10% > HCC out of samples with H-scores in both HCC and TAT tissue	65% of 69	78% of 67
Percentages of samples with TAT 10% > HCC out of samples with HCC < TAT	88% of 51	93% of 56

### Statistical analysis of TGFBR1 and TGFBR2 staining intensity as biomarkers for HCC diagnosis

Using the HCC tissue and cirrhosis-only tissue results from the discovery set and the subset of samples in validation set 2 that were labelled at GW (a total of 81 patient samples with H- scores), we calculated a mean H-score of 182.0 ± 57.7 (SD) for TGFBR1 and of 124.6 ± 73.0 for TGFBR2 across diagnostic groups. Both were normally distributed without outliers (Figure 3A). Comparing the mean H-scores for patients with cirrhosis only versus those with HCC, we found that staining for both TGFBR1 and TGFBR2 was slightly but not significantly higher for the patients with cirrhosis only (TGFBR1 mean 196.1 ± 44.6 versus 174.6 ± 62.6, *p* = 0.10; TGFBR2 mean 145.2 ± 78.9 versus 113.7 ± 67.9, *p* = 0.06). Based on these results, we tested H- scores for TGFBR1 ranging from 140 to 200 as thresholds for predicting cirrhosis only versus HCC (Table 2) and we tested H-scores ranging from 114 to 145 as TGFBR2 H-score thresholds (Table 3). H-score thresholds that performed best were 190 for TGFBR1 with sensitivity 0.58, specificity 0.50, and overall prediction accuracy of 0.56; and 130 for TGFBR2 with sensitivity 0.62, specificity 0.61, and overall accuracy 0.62.

**Table 2:** Predictive power of TGFBR1 labeling intensity in correctly predicting the presence of cirrhosis only

	Threshold						
**Value**	140	150	160	170	180	190^**a**^	200
**Sensitivity**	0.30 (0.18–0.43)	0.30 (0.18–0.43)	0.40 (0.26–0.53)	0.47 (0.34–0.61)	0.55 (0.41–0.68)	0.58 (0.45–0.72)	0.62 (0.49–0.75)
**Specificity**	1 (1–1)	0.89 (0.78–1.01)	0.79 (0.63–0.94)	0.71 (0.55–0.88)	0.50 (0.31–0.69)	0.50 (0.31–0.69)	0.46 (0.28–0.65)
**Positive predictive value**	1 (1–1)	0.84 (0.68–1.01)	0.78 (0.62–0.93)	0.76 (0.61–0.90)	0.67 (0.53–0.81)	0.69 (0.55–0.82)	0.69 (0.56–0.82)
**Negative predictive value**	0.43 (0.31–0.55)	0.40 (0.28–0.53)	0.41 (0.28–0.54)	0.42 (0.28–0.56)	0.37 (0.22–0.52)	0.39 (0.23–0.55)	0.39 (0.23–0.56)
**Accuracy**	0.54 (0.43–0.65)	0.51 (0.40–0.62)	0.53 (0.42–0.64)	0.56 (0.45–0.66)	0.53 (0.42–0.64)	0.56 (0.45–0.66)	0.57 (0.46–0.68)

^a^Optimal threshold.

**Table 3:** Predictive power of TGFBR2 labeling intensity in correctly predicting the presence of cirrhosis only

	Threshold						
**Value**	115	120	125	130	135	140^a^	145
**Sensitivity**	0.51 (0.37–0.64)	0.51 (0.37–0.64)	0.55 (0.41–0.68)	0.62 (0.49–0.75)	0.7 (0.57–0.82)	0.7 (0.57–0.82)	0.72 (0.6–0.84)
**Specificity**	0.61 (0.43–0.79)	0.61 (0.43–0.79)	0.61 (0.43–0.79)	0.61 (0.43–0.79)	0.54 (0.35–0.72)	0.54 (0.35–0.72)	0.54 (0.35–0.72)
**Positive predictive value**	0.71 (0.57 –0.85)	0.71 (0.57 –0.85)	0.73 (0.59 –0.86)	0.75 (0.62–0.88)	0.74 (0.62–0.86)	0.74 (0.62–0.86)	0.75 (0.63–0.86)
**Negative predictive value**	0.4 (0.25 – 0.54)	0.4 (0.25–0.54)	0.41 (0.26 –0.57)	0.46 (0.3–0.62)	0.48 (0.31–0.66)	0.48 (0.31–0.66)	0.5 (0.32–0.68)
**Accuracy**	0.54 (0.43–0.65)	0.54 (0.43–0.65)	0.57 (0.46–0.68)	0.62 (0.51–0.72)	0.64 (0.54–0.75)	0.64 (0.54–0.75)	0.65 (0.55–0.76)

^a^Optimal threshold.

Next, we used multivariable logistic regression to fit a model using H-scores from both TGFBR1 and TGFBR2 scores with HCC versus cirrhosis as the dependent variable. Neither TGFBR1 nor TGFBR2 had a significant independent contribution to the model for predicting if a patient had cirrhosis only versus HCC: The TGFBR1 adjusted odds ratio (aOR) for cirrhosis versus HCC = 1.006 [95% confidence interval (CI): 0.997–1.016], *p* = 0.21; TGFBR2 aOR = 1.005 [95% CI: 0.998–1.013], *p* = 0.16). The model had an area under the receiver operating curve (AUROC) of 0.67.

Using the regression model to calculate each patient’s probability of cirrhosis, we found that the mean probability of a patient having cirrhosis only differed between the diagnostic groups (0.41 [95% CI: 0.35–0.46] in cirrhosis alone versus 0.33 [95% CI: 0.30–0.37] in HCC (*p* = 0.017). We examined various cut points in the probability distribution for having HCC and determined the model’s sensitivity, specificity, and overall prediction accuracy at each probability threshold. At a probability level of 0.4, sensitivity was 0.76, specificity was 0.61, and overall accuracy 0.71 (Table 4). We assessed the calibration of the logistic regression model by dividing the probability distribution into quintiles and found that there was moderately good correspondence between the predicted probability of having cirrhosis only and the observed incidence (R^2^ = 0.62; linear equation y = 1.08 × –0.03) (Figure 3B).

**Table 4:** Predictive power of the model that incorporates both TGFBR1 and TGFBR2 staining intensity for predicting the presence of cirrhosis only

	Threshold						
**Value**	0.30	0.35	0.37	0.39	0.4^a^	0.45
**Sensitivity**	0.4 (0.26–0.54)	0.6 (0.46–0.74)	0.64 (0.51–0.77)	0.72 (0.60–0.84	0.76 (0.64–0.88)	0.84 (0.74–0.94)
**Specificity**	0.71 (0.55–0.88)	0.64 (0.47–0.82)	0.61 (0.43–0.79)	0.61 (0.43–0.79)	0.61 (0.43–0.79)	0.39 (0.21–0.57)
**Positive predictive value**	0.71 (0.55–0.88)	0.75 (0.62–0.88)	0.74 (0.61–0.87)	0.77 (0.64–0.89)	0.78 (0.66–0.89)	0.71 (0.60–0.83)
**Negative predictive power**	0.40 (0.26–0.54)	0.47 (0.31–0.63)	0.49 (0.32–0.65)	0.55 (0.37–0.72)	0.59 (0.41–0.77)	0.58 (0.36–0.80)
**Accuracy**	0.51 (0.40–0.62)	0.62 (0.51–0.72)	0.63 (0.52–0.74)	0.68 (0.58–0.78)	0.71 (0.60–0.81)	0.68 (0.58–0.78)

^a^Optimal threshold.

## DISCUSSION

We found a significant reduction in TGFBR2 in tissue from patients with HCC compared with tissue from patients with only cirrhosis and that this reduction consistently occurred in regions near tumor tissue in patients with HCC. Furthermore, we established that this reduction was consistent across samples obtained from cohorts of patients at 3 institutions representing diverse etiologies and demographic groups. We also determined that only a subset of the patient cohorts exhibited a consistent significant reduction in TGFBR1 intensity both within a patient sample in regions near tumor tissue in patients with HCC and between patients when comparing those with HCC to those with only cirrhosis. Automated image analysis confirmed the findings. Analysis of HCC and TAT from the same slide showed a high frequency of reduced TGFBR1 and TGFBR2 staining in the HCC tissue with this relative difference most consistently found with TGFBR2 staining.

From these findings, we developed models based on various intensities of TGFBR2 staining and tested their ability diagnostically differentiate HCC from cirrhosis. Unfortunately, TGFBR2 staining intensity, alone or in combination with TGFBR1 staining intensity, was sufficiently variable that no threshold of staining intensity had sufficient predictive power to be used as a diagnostic for HCC. However, integration of such data into more complex models could improve diagnostic power.

Although our analysis indicated that a simple value of TGFBR2 staining intensity is insufficient to detect HCC in the context of cirrhosis, our findings indicated that a relative reduction in staining of TGFBR2 in the context of the high staining associated with cirrhotic tissue is an indication of HCC. Furthermore, an AI-based image analysis reliably detected this reduction. The ability to generate an AI-based image analysis pipeline for quantitative IHC has implications for studies involving samples acquired and processed at multiple institutions. Biopsy tissue can provide clues to understanding the molecular pathology of the disease and guide enrollment of patients in clinical trials [27]. Because multiple sites often enroll patients in clinical trials, ensuring standardized sample processing and image analyses are critical to producing comparable results across cohorts of patients treated at different locations and different times. Our data showed that even with a protein with high variability, both within a biopsy and across patient samples, an AI-based automated image analysis pipeline could effectively provide a quantitative assessment of IHC results in liver biopsy tissue. Although our study found a low error rate for the AI-based pipeline based on the evaluation of a single pathologist, future studies are needed to confirm that this AI-based approach meets or exceeds the accuracy of sample evaluation by multiple pathologists. Such validated pipelines can overcome challenges in the use of tissues from multiple sites, which are associated with variabilities in tissue processing, antibody staining, and interpretations of histopathology.

Although most proteins that serve as biomarkers typically increase in the target condition, decreases in protein abundance are also indicators of disease. Indeed, differences in the activity of the TGF-β pathway are associated with the outcome of HCC patients. Analysis of genetic alterations in HCC reveal that some patients have a profile of activated TGF-β signaling and others with a profile of inactivated TGF-β signaling. Those with the inactivated TGF-β pathway signature have a worse prognosis [28]. Another study classified HCC patients with a profile that depended on TGFBR2 into two groups and the group with the “late” TGF-β signature had worse prognosis [29]. Thus, the ability to quantify differences in TGFBR2 has prognostic value.

Our findings indicated that reduced TGFBR2 abundance relative to that in the surrounding tissue is a biomarker for HCC in cirrhotic tissue. The data suggested that reduced TGFBR2 rather than TGFBR1 represents a more common mechanism for the loss of TGF-β signaling in HCC. Furthermore, the HCC-associated reduction in TGFBR2 is consistent with loss of the tumor-suppressor function of TGF-β signaling in hepatocytes [22].

Individuals at high risk of developing HCC benefit from regular surveillance by abdominal ultrasound. In cases where the suspicious nodules cannot be conclusively classified as HCC from non-invasive imaging, biopsy remains the standard. However, it can be difficult to differentiate between well and poorly differentiated HCC from morphological properties alone. IHC for biomarkers, such as GPC3, HSP70, ARG1 (Arginase 1), CD34, and GS, is used to improve accuracy [27]. For example, GPC3 positivity is associated with poorly differentiated tumors [30]. However, no single biomarker or morphological property is sufficient to diagnose high-grade dysplastic nodules, well-differentiated HCC, and poorly differentiated HCC [31]. Future studies are needed to address if the reduction in TGFBR2 is associated with precancerous nodules or with well-differentiated or poorly differentiated HCC. Our findings support further analyses to determine if this reduction is an early occurrence that can be used diagnostically in combination with other morphological characteristics or biomarkers to detect HCC at early stages in high-risk patients.

## METHODS

### Study design and participants

This multi-institutional study aimed to determine if differences in the abundance of the receptors for TGF-β were useful as biomarkers that could differentiate HCC from cirrhotic tissue in patient tissue samples. Tissue samples were obtained from pathology biorepositories at GW, UMD, and UH. IHC was performed on tissue samples for TGFBR1 and TGFBR2. Logistic regression modeling was used to correlate protein abundance with clinical attributes. An automated image analysis was developed with AIFORIA. The study included samples from 97 patients with HCC (including 1 HCC case from the cirrhosis group who developed HCC in the follow-up period), and from 53 cirrhosis patients without HCC for AI-based image analysis. Because the samples were from biorepositories, this study qualified for institutional review board exemption. Before processing the samples, all personal identification information and unique identifiers were replaced with project-specific codes.

### Properties of biorepository specimens, including demographic and clinical data of HCC and cirrhosis-only cohorts

The HCC cohorts consisted of patients with pathologically diagnosed HCC per established histologic criteria. Pathological specimens of HCC were obtained from tissue taken from patients either undergoing hepatic resection or explant samples in patients who had undergone LT. In those who had undergone locoregional therapy prior to LT, HCC samples were selected from viable, non-necrotic tissue from 37 specimens at GW, 43 specimens at UMD, and 18 specimens at UH. The cirrhosis cohorts consisted of patients with cirrhosis without HCC who underwent LT or liver biopsy. Cirrhosis-only specimens were from GW (8 specimens) and UMD (45 specimens). Clinical and demographic characteristics were collected, and the number of tissue samples with quantifiable staining were recorded (Table 5). Clinical data included the following etiologies: viremic or cured hepatitis virus C (HCV) infection, hepatitis B virus (HBV) infection, alcohol-associated liver disease, and non-alcoholic fatty liver disease (NAFLD). Etiologies were non-exclusive.

**Table 5:** Clinical and demographic characteristics of patients from each site and HCC or cirrhosis status of samples with TGFBR1 and TGFBR2 staining

Patient information	GW (*n* = 47)	UH (*n* = 18)	UMD (*n* = 88)
Median age	60	65	58.5
**Sex, *n* (%)**			
Male	37 (78.7)	15 (83.3)	61 (69.3)
Female	10 (21.3)	3 (16.7)	27 (30.7)
**Race (%)**			
White	31.9%	16.7%	64.8%
Black	34.0%	5.6%	23.9%
Asian	31.9%	44.4%	2.2%
Mixed or other	2.2%	33.3%	9.1%
**TGFBR1 or TGFBR2 staining, *n*/total (%)^a^**			
HCC for TGFBR1	37/45 (82.2)	18/18 (100)	42/86 (48.8)
Cirrhosis for TGFBR1	8/45 (17.8)	0	44/86 (51.2)
HCC for TGFBR2	35/43 (81.4)	18/18 (100)	43/88 (48.9)
Cirrhosis for TGFBR2	8/43 (18.6)	0	45/88 (51.1)
HCC for TGFBR2	35/43 (81.4)	18/18 (100)	43/88 (48.9)
Cirrhosis for TGFBR2	8/43 (18.6)	0	45/88 (51.1)
**Main etiologies (%)^b^**			
HCV, viremic	36.2	0	20.4
HCV, cured	4.3	22.2	11.4
HBV	31.9	5.6	1.1
Alcohol	44.7	22.2	36.4
NAFLD	25.5	11.1	13.6

^a^Some samples excluded due to poor quality of the tissue section. ^b^Some patients presented with more than one etiology.

### Immunohistochemistry

All samples were formalin-fixed paraffin-embedded (FFPE). Samples were divided into sets for TGFBR1 and TGFBR2 staining. Samples with mixed cholangiocarcinoma were excluded from manual IHC analysis but were included for automated image analysis. Slides were deparaffinized in xylene followed by rehydration through graded alcohol and finally rinsed in distilled water. Heat-induced antigen retrieval was performed with citrate buffer (pH 6.0, #H-3300, Vector Labs) or EDTA solution (pH 9.0, #H-3301, Vector Labs) for 25 minutes at 95°C. Endogenous peroxidase activity was blocked with 3% H2O2 in methanol at room temperature. Subsequently, tissue sections were blocked with goat serum and stained with primary antibodies at room temperature for an hour. Primary antibodies recognized TGFBR1 (#31013, Abcam) or TGFBR2 (#61213, Abcam). Antibody binding was detected with biotinylated secondary goat anti-rabbit antibodies and ABC solution from the anti- rabbit kit (#PK-61-1, Vector Laboratories) followed by the EnVision^™^ FLEX Substrate Working Solution (Dako) for five minutes at room temperature. All sections were counterstained with hematoxylin, dehydrated in ethanol and xylene, and coverslipped. Staining was performed at GW, UMD, or UH using reagents and the protocol provided by GW.

Samples from HCC patients were classified into regions of HCC tissue and TAT or as mixed HCC and cholangiocarcinoma. Samples from patients without HCC were classified as cirrhosis-only tissue. Pathologists at GW, blinded to the classification, interpreted the TGFBR1 and TGFBR2 staining. Samples were assigned an H-score between 0 and 300 for TGFBR1 and TGFBR2 staining intensities [32]. H-score was determined by assigning each cell a staining intensity of 0 for no signal, 1 for weak signal, 2 for mild signal, and 3 for strong signal; then the percentage of cells at each intensity score was weighted by the intensity score and the sum of these weighted values were calculated:

H-score = 3 × (% of strongly labeled cells) + 2 × (% of moderately labeled cells) + 1 × (% of weakly labeled cells) + 0 × (% of cells with no labeling).

Samples that contained mixed HCC and cholangiocarcinoma or samples that were distorted or damaged were not quantified. Differences between mean H-scores of TGFBR1 or TGFBR2 in HCC tissue and TAT or in HCC tissue and cirrhosis-only tissue were evaluated by two-tailed paired *t*-test.

H-scores for TGFBR1 and TGFBR2 staining were compared within samples for regions of HCC tissue and TAT. A threshold of a 10% difference in H-score was used to compare differences in staining within individual samples from patients with HCC.

### Development of AI-based image analysis

Slides labelled with TGFBR1 or TGFBR2 were digitized using a Panoramic P1000 whole slide scanner (3DHistech, Budapest, Hungary) at 20×. Images were stored in a proprietary format (NDPI) with a pixel in the image corresponding to a 0.227 × 0.227 μm area in the sample.

Images were compressed to a JPEG file format (Enhanced Compressed Wavelet, ECW, ER Mapper; Intergraph, Atlanta, Georgia, USA) with a quality factor of 80. The compressed virtual slides were uploaded to a whole-slide image management server (WebMicroscope; Fimmic Oy, Helsinki, Finland).

For the digital image analysis of the samples, image analysis software platform (WebMicroscope) was used. This platform uses deep learning–based machine-learning algorithms to create computer vision applications. The model for the differentiation of HCC and cirrhotic tissues consists of two algorithms. The first algorithm analyzes the whole image to identify and quantify the area of the regions of HCC within the sample and then quantifies the percentage area representing different intensity of labeling. The second algorithm detects the regions of cirrhotic tissue and quantifies the percentage area of different intensity of labeling.

To train the algorithm to recognize cirrhotic tissue from HCC tissue, the training set consisted of whole-slide images from 20 patients that were selected on a case-by-case basis to ensure that the slide contained mostly tumor or cirrhotic tissue without large regions of necrotic tissue. Slides with areas defined as cirrhosis were from samples from individuals with cirrhosis and without a diagnosis of HCC. Slides with areas defined as HCC were from individuals diagnosed with HCC. The output of this algorithm is the “parent layer,” representing cirrhotic, HCC, or adjacent tissue. Using these 20 selected slides, a second algorithm was trained to identify arbitrarily defined intensities of IHC labeling: negative, weak, medium, and strong. The output of this algorithm is the “intensity layer,” which is then integrated with the parent layer to define labeling intensity for TGFBR1 or TGFBR2 within the areas defined as cirrhotic, HCC, or TAT. Separate models were developed and trained for TGFBR1 and TGFBR2.

Assessment was performed in >40% patient slides to assure the sensitivity and specificity of the system. For each sample, the H-scores were calculated for both manual and AI-based results and compared. The TGFBR1 AI model was tested using 71 whole-slide images for the parent layer with annotations totaling 302.48 mm^2^ and 17 whole-slide images for the intensity layer with annotations totaling 20.41 mm^2^. From a random set of 40% of the samples, the pathologist validated the AI-based analysis for the accuracy of the annotation of different regions (cirrhosis, HCC, or TAT) in the same slide by the algorithms used. The percentage of inaccurate annotations in the AI-based analysis (those deemed incorrect by the pathologist) were 1.82% for the parent layer and 3.08% for the intensity layer. The TGFBR2 AI model was trained using 45 whole-slide images for the parent layer with annotations totaling 223.26 mm^2^ and 18 whole-slide images for the intensity layer with annotations totaling 37.31 mm^2^. The error in the AI-based annotations based on a randomly selected 40% of images evaluated by the pathologist was 0.92% for the parent layer and 1.28% for the intensity layer.

### Analysis of the diagnostic value of manually assigned TGFBR1 and TGFBR2 H-scores

The distribution of the intensity of staining for TGFBR1 and TGFBR2 in samples from the discovery set and validation set 2 labelled at GW were checked for outliers and non-normality. Significant differences in the means of the H-scores between diagnostic groups (HCC or cirrhosis) were determined using two-tailed between-group *t*-test.

To assess the diagnostic usefulness of TGFBR1 or TGFBR2 labeling intensity in differentiating HCC from cirrhosis, we evaluated a range of H-score thresholds for each marker, testing the sensitivity, specificity, positive predictive value, negative predictive value, and total prediction accuracy for detecting cirrhosis only versus HCC at each threshold. We used multivariable logistic regression to create a prediction model for HCC versus cirrhosis only that incorporated both TGFBR1 and TGFBR2 labeling intensity:

Risk = intercept + beta1 × (TGFBR1 H-score) + beta2 × (TGFBR2 H-score).

The final model was as follows:

Risk = −2.4106 + 0.00619 × (TGFBR1 H-score) + 0.00543 × (TGFBR2 H-score).

This model was used to calculate the probability of having cirrhosis only versus HCC for each subject for whom both TGFBR1 and TGFBR2 was detectable:

Prob(Cirrhosis) = exp(risk)/(1 + exp(risk)).

Calibration of this model was assessed by comparing observed versus predicted cirrhosis only versus HCC diagnosis within quintiles of the probability distribution. For perfect calibration, we expect a slope of 1, and an intercept of 0, with the points spread across the full range of probabilities from 0% to 100%.

Statistical tests used SAS (Version 9.4, Cary, NC) with *p* < 0.05 considered significant.

## SUPPLEMENTARY MATERIALS



## Abbreviations

HCC: hepatocellular carcinoma
NAFLD: non-alcoholic fatty liver disease
HCV: hepatitis C virus
HBV: hepatitis B virus
TGF-β: transforming growth factor β
TGFBR1: TGF-β receptor 1
TGFBR2: TGF-β receptor 2
