# Supplementary material for: Using quantitative immunohistochemistry in patients at high risk for hepatocellular cancer
Source: Genes Cancer. 2022 Jun 6;13:9–20. doi: 10.18632/genesandcancer.220 (PMC9170384; doi:10.18632/genesandcancer.220)
Supplement: Supplementary file 1 [file ganc-13-220-s001.pdf]

Using quantitative immunohistochemistry in patients at high risk for hepatocellular cancer

SUPPLEMENTARY MATERIALS

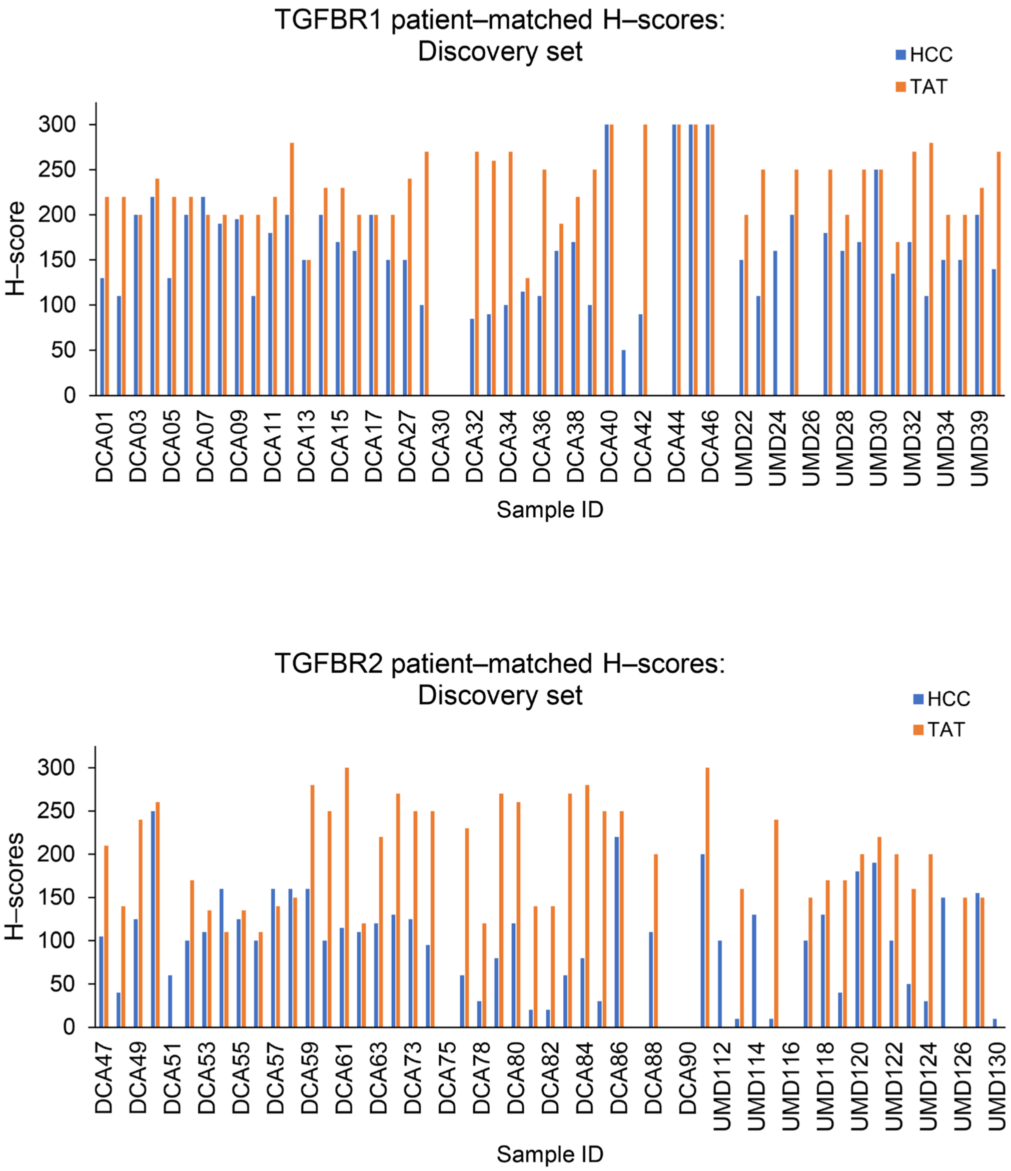

Supplementary Figure 1: Patient-matched H-scores for HCC and tumor-adjacent tissue from the discovery set.

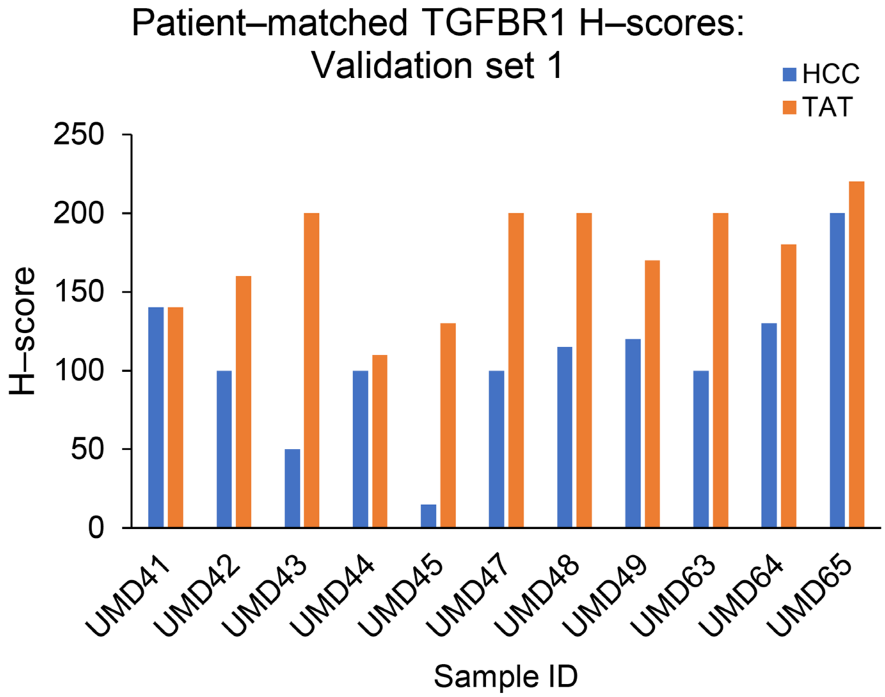

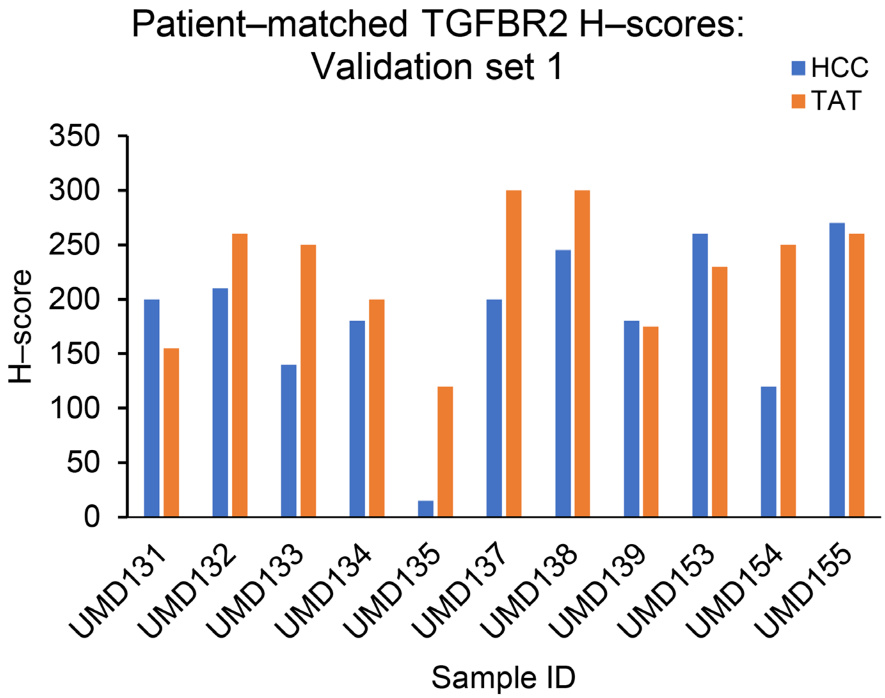

Supplementary Figure 2: Patient-matched H-scores for HCC and tumor-adjacent tissue from validation set 1.

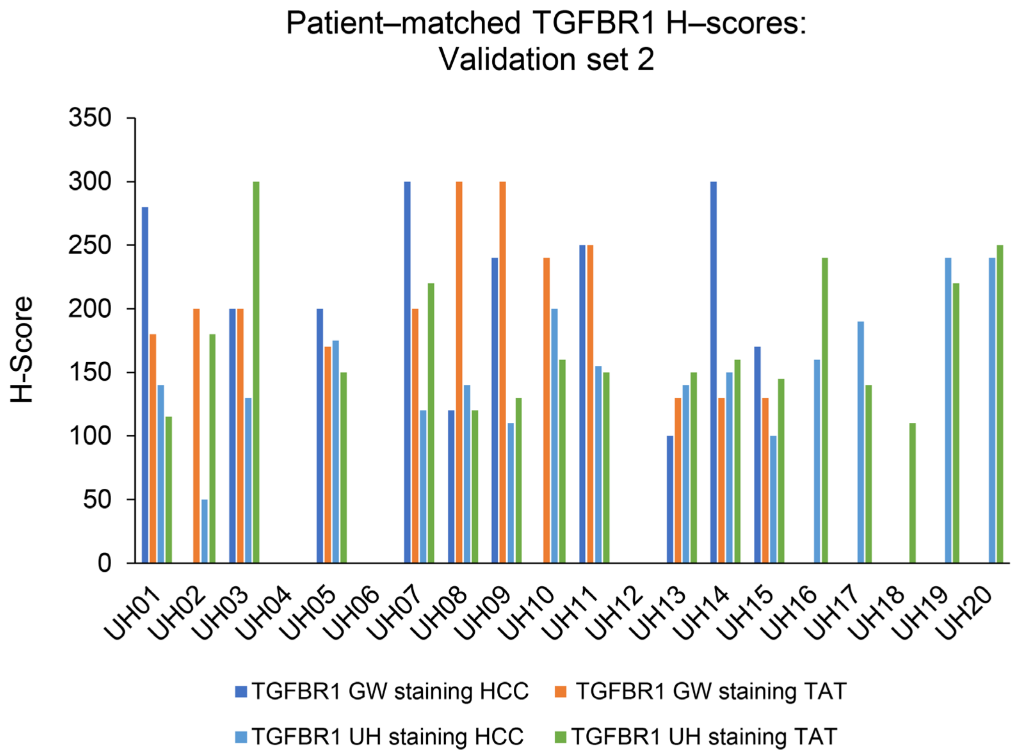

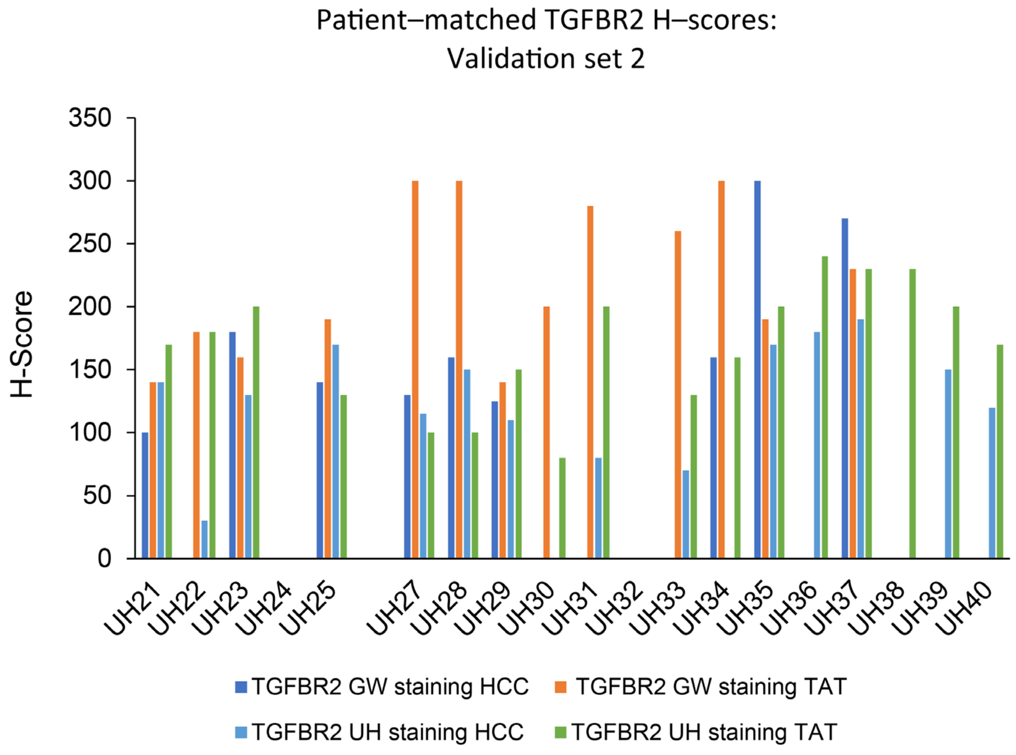

Supplementary Figure 3: Patient-matched H-scores for HCC and tumor-adjacent tissue from validation set 2.
